# Effects of ocean acidification on the potency of macroalgal allelopathy to a common coral

**DOI:** 10.1038/srep41053

**Published:** 2017-02-01

**Authors:** Carlos Del Monaco, Mark E. Hay, Patrick Gartrell, Peter J. Mumby, Guillermo Diaz-Pulido

**Affiliations:** 1 Griffith School of Environment & Australian Rivers Institute - Coast & Estuaries, Nathan Campus, Griffith University, 170 Kessels Road, Brisbane, Nathan, Queensland 4111, Australia; 2 School of Biology, and Aquatic Chemical Ecology Center, 950 Atlantic Dr., Georgia Institute of Technology, Atlanta, Georgia, 30332, USA; 3 Marine Spatial Ecology Lab, School of Biological Sciences, The University of Queensland, St Lucia, Queensland 4072, Australia; 4 Australian Research Council Centre of Excellence for Coral Reef Studies, St Lucia, Australia.; 5 Australian Research Council Centre of Excellence for Coral Reef Studies, Townsville, Australia.

## Abstract

Many coral reefs have phase shifted from coral to macroalgal dominance. Ocean acidification (OA) due to elevated CO_2_ is hypothesised to advantage macroalgae over corals, contributing to these shifts, but the mechanisms affecting coral-macroalgal interactions under OA are unknown. Here, we show that (i) three common macroalgae are more damaging to a common coral when they compete under CO_2_ concentrations predicted to occur in 2050 and 2100 than under present-day conditions, (ii) that two macroalgae damage corals via allelopathy, and (iii) that one macroalga is allelopathic under conditions of elevated CO_2_, but not at ambient levels. Lipid-soluble, surface extracts from the macroalga *Canistrocarpus* (=*Dictyota) cervicornis* were significantly more damaging to the coral *Acropora intermedia* growing in the field if these extracts were from thalli grown under elevated vs ambient concentrations of CO_2_. Extracts from the macroalgae *Chlorodesmis fastigiata* and *Amansia glomerata* were not more potent when grown under elevated CO_2_. Our results demonstrate increasing OA advantages seaweeds over corals, that algal allelopathy can mediate coral-algal interactions, and that OA may enhance the allelopathy of some macroalgae. Other mechanisms also affect coral-macroalgal interactions under OA, and OA further suppresses the resilience of coral reefs suffering blooms of macroalgae.

Coral reefs are one of the most diverse and complex ecosystems on the planet and provide livelihoods, food, and important ecosystem services for hundreds of millions of people^1^. However, a large proportion of reefs worldwide are severely degraded, and many reefs are on a trajectory of decline^2^,^3^. A symptom of decline is a reduced cover of reef-building corals and an increased abundance of upright macroalgae^4^,^5^. Understanding the drivers of macroalgal increases and the effects of coral-macroalgal interactions on reef degradation and resilience is of critical importance for the conservation of reef ecosystems^6^,^7^,^8^.

The drivers of coral loss and macroalgal increase are varied and include herbivore loss through overfishing or disease, decreased water quality associated with nutrient enrichment and sedimentation, coral mortality caused by predators, hurricanes and cyclones, bleaching and diseases, algal colonization associated with coral morality, and a failure of juvenile corals and fishes to recruit once reefs become algal dominated^2^,^9^,^10^,^11^,^12^. Problems arising from human-induced ocean acidification, that occurs as atmospheric CO_2_ is absorbed by the ocean and lowers pH^13^, have also been suggested as potential drivers of elevated algal populations on reefs^14^,^15^,^16^. Elevated seawater CO_2_ concentrations may enhance fleshy algal growth rates^15^,^17^,^18^, reduce coral growth and calcification rates^19^, and strengthen space competition between macroalgae and corals, with outcomes that favour macroalgae over corals^14^,^15^,^20^. Habitats with naturally elevated CO_2_ concentrations show increased fleshy macroalgal abundance^21^ and decreased coral cover compared to control sites^16^, suggesting shifts in competitive interactions between taxa under conditions of ocean acidification. Manipulative experiments on the role of elevated CO_2_ in coral-macroalgal competition are currently limited to a single pair of species^15^ and it is unclear whether this coral-algal pairing is representative of such interactions. Moreover, the underlying mechanism for altered competitive ability has not been identified.

Some algal competitive mechanisms have been recognised. Macroalgae may directly overgrow coral tissue, reduce light levels required for photosynthesis, abrade tissue, or produce chemical compounds that directly^6^,^22^,^23^,^24^or indirectly stress or kill corals^25^,^26^. Macroalgae may also harm corals by modifying coral microbial community composition^27^,^28^. However, the effect of ocean acidification on the potency of algal allelopathy to corals is uninvestigated.

In terrestrial systems, elevation of CO_2_ tends to increase the production of plant secondary metabolites, but responses are variable^29^,^30^. The limited investigations in marine systems suggest that elevated CO_2_ decreases phenolics in seagrasses^31^ and brown macroalgae^32^ or has little effect on terpenes in calcareous algae^33^. Elevated CO_2_ could increase the production and release of macroalgal primary metabolites (dissolved organic carbon -DOC), and release of DOC has been associated with a detrimental increase in microbial activity and hypoxic conditions on coral surfaces^25^,^34^, however, water flow may limit hypoxic conditions at coral-algal interfaces^35^,^36^. At present, effects of ocean acidification on chemically-mediated coral-macroalgal interactions are unknown.

Because most macroalgal secondary metabolites are carbon-based^37^,^38^,^39^, we evaluate the hypothesis that ocean acidification might intensify the potency of allelopathic interactions, strengthen the competitive abilities of macroalgae over corals, contribute to coral mortality, and possibly exacerbate coral-to-algal phase shifts. We use a combination of outdoor tank and *in-situ* field experiments to quantify the effects of elevated CO_2_ on the strength of allelopathic interactions between macroalgae and a common coral on the Great Barrier Reef (GBR), Australia. We assess the allelopathic potency, rather than the concentrations, of extracts from the surfaces of macroalgae because many allelopathic compounds are unknown, may act synergistically or additively and thus their combined effects cannot be quantified by evaluating the concentrations of a few known compounds. To examine the generality of macroalgal effects, we used three species of macroalgae (seaweeds) common in GBR reefs: *Chlorodesmis fastigiata, Canistrocarpus cervicornis* (synonym *Dictyota cervicornis*) and *Amansia glomerata* and tested the impact of each on the common coral *Acropora intermedia.*

First, we conducted an experiment to assess competitive outcomes between the coral *A. intermedia* and each macroalga when in direct contact and exposed to either present day levels of CO_2_ or to levels projected to occur by the mid and end of the century under the RCP 8.5 CO_2_ emission scenarios (ambient = 380 CO_2_ ppm; mid-century = 540 CO_2_ ppm; end-of-century = 936 CO_2_ ppm, see Methods). Once the ability of macroalgae to damage coral tissue under elevated CO_2_ levels was established, we conducted a second experiment to assess the effect of algal allelochemicals alone by embedding natural concentrations of lipid-soluble extracts from algal surfaces into experimental gels and applying these, and appropriate controls, to *A. intermedia* growing in the field. For this experiment, the three species of macroalgae were incubated (in isolation) under either high CO_2_ concentrations (936 CO_2_ ppm) or ambient CO_2_ concentrations (380 CO_2_ ppm) and their lipid-soluble, surface chemicals extracted at the end of the 14 day incubation period. Although macroalgae may induce allelopathy when competing with corals^40^ we incubated the algae in isolation to evaluate the effects of elevated CO_2_ alone. Surface extracts were then imbedded in gel pads that were placed in contact with un-manipulated corals *in situ* on the reef. We used un-manipulated corals to avoid confounding altered potency of the extracts with changed susceptibility of the corals exposed to differing levels of CO_2_. The chlorophyll fluorescence of the coral’s symbiotic dinoflagellates, which indicates the overall photosynthetic efficiency of corals, was assessed as an indicator of stress^41^ caused by macroalgal allelopathy^22^,^25^,^38^.

## Results

### Effects of CO_2_ and coral-macroalgal competition on coral tissue loss (partial morality)

**Figure 1:**
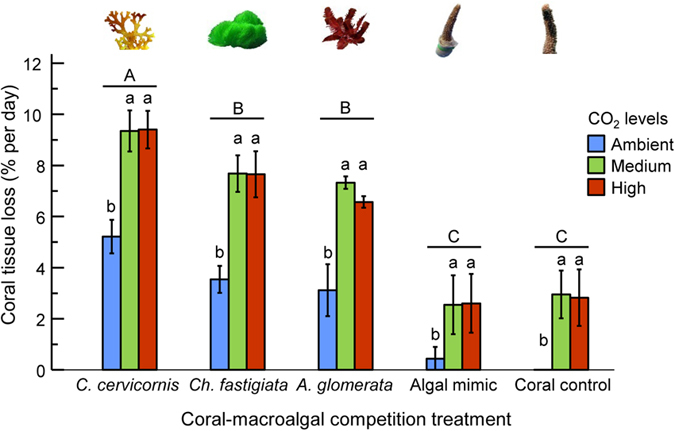
Mean rate of tissue loss (percent per day) in *Acropora intermedia* corals exposed to varying levels of ocean acidification and coral-macroalgal competition treatments. The rate was calculated by estimating the amount of coral tissue loss per day and values were then averaged by the number of days until complete loss of coral tissue. N = 12 coral branches (±SEM). Corals were exposed to contact with the macroalgae *Canistrocarpus* (=*Dictyota) cervicornis, Chlorodesmis fastigiata, Amansia glomerata* or a plastic mimic under three levels of CO_2_: ambient (380 ppm); medium (540 ppm) or high (936 ppm). Controls were corals without macroalgal contact. Data were analysed with a two-way ANOVA. Lowercase (a,b) and uppercase (A,B,C) letters indicate significant differences among CO_2_ and competition treatments, respectively, via Tukey tests. Complete ANOVA and Tukey test results are in Table 1.

**Figure 2:**
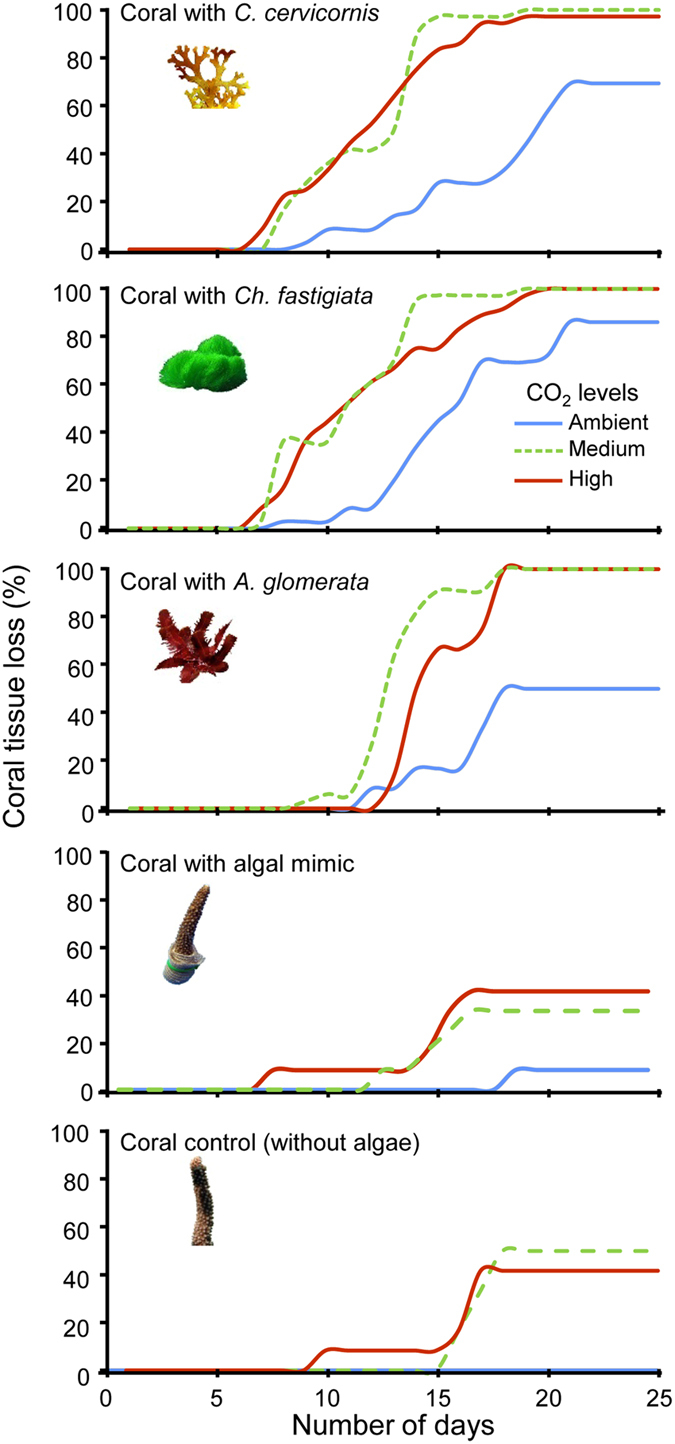
Percentage of coral tissue loss through time during a coral-algal competition and ocean acidification experiment. *Acropora intermedia* corals were exposed to contact with *Canistrocarpus* (=*Dictyota) cervicornis, Chlorodesmis fastigiata, Amansia glomerata* or a plastic mimic under three levels of CO_2_: ambient (380 ppm); medium (540 ppm) or high (936 ppm). Controls were corals without macroalgal contact. Coral tissue loss was estimated daily by counting the number of coral branches that exhibited any tissue loss and data are presented as frequency of dead colonies over the experimental period (25 days).

The coral-macroalgal competition and CO_2_ treatments significantly affected coral tissue loss. Macroalgal contact with corals increased the rate of coral tissue loss in all CO_2_ conditions (Fig. 1, Table 1), but the rate of tissue death per day increased significantly under elevated concentrations of CO_2_ (Figs 1 and 2, Table 1). Coral tissue death also varied by algal species, with *C. cervicornis* being strongly and significantly more damaging than *A. glomerata*. Corals exposed to any macroalga experienced significantly higher partial mortality (Table 1), and experienced it more rapidly, than control corals or corals exposed to the algal mimic (Fig. 2). This suggests a chemical or biological mechanism rather than simple impacts due to shading or abrasion. However, corals isolated from all macroalgae and corals in contact with plastic macroalgal mimics (mimicking the physical presence of algae) exposed to medium or high CO_2_ levels also experienced some tissue loss, indicating that OA alone led to some coral damage (Figs 1 and 2).

### Effects of CO_2_ on the potency of macroalgal allelochemicals

**Figure 3:**
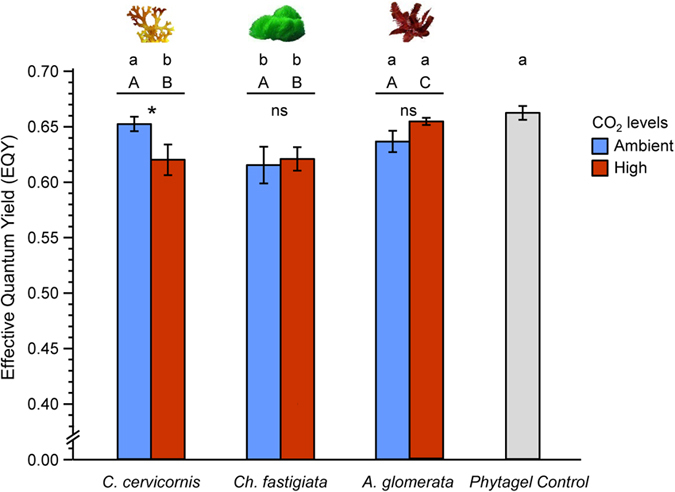
Mean effective quantum yield (±SEM, n = 15) of symbiotic dinoflagellates in *in-situ* corals exposed to contact with lipid-soluble surface extracts from the macroalgae *Canistrocarpus* (=*Dictyota) cervicornis, Chlorodesmis fastigiata* and *Amansia glomerata* grown under elevated CO_2_ (936 CO_2_ ppm) and ambient conditions (380 ppm). Control corals were in contact with gel pads treated with carrier solvent but without inclusion of algal extract (i.e. Phytagel controls). Extract from *C. cervicornis* grown under enriched CO_2_ was significantly more suppressive of photosynthetic efficiency (EQY) compared to extracts from *C. cervicornis* grown under ambient CO_2_. Elevated CO_2_ did not affect the potency of extracts from the other two algal species. Data were analysed with a two-way ANOVA: Asterisks and uppercase (A,B,C) letters indicate significant differences among CO_2_ and macroalgal treatments, respectively, via posthoc Tukey tests (*p < 0.05, ns: not significant). Complete ANOVA and Tukey test results are in Table 2. Lowercase letters (a,b) indicate significant differences in EQY of the coral compared to Phytagel controls via Dunnett test. Note y-axis break.

Elevated CO_2_ enhanced macroalgal allelopathy against *A. intermedia* but only for the alga *C. cervicornis* (Fig. 3, Table 2). Allelochemicals from *C. cervicornis* incubated under high CO_2_ conditions suppressed effective quantum yield (EQY) significantly more than extracts from thalli grown in ambient CO_2_ (p = 0.037, Table 2). Thus, for *C. cervicornis*, elevated CO_2_ concentrations enhanced the negative effects of allelochemicals on coral tissue. In contrast, surface extracts from *Ch. fastigiata* and *A. glomerata* were not significantly more potent when grown under elevated CO_2_ concentrations (Fig. 3, Table 2). The allelopathic effect on corals varied among macroalgal species, with surface extracts from *Ch. fastigiata* [grown under both ambient and high CO_2_ (Dunnett test, p = 0.007 and 0.022, respectively)] and *C. cervicornis* (only under high CO_2_) significantly reducing effective quantum yield relative to control treatments (Phytagel controls, i.e. gel pads without allelochemicals) (Fig. 3). Extracts from *A. glomerata* did not cause significant reduction in effective quantum yield at any CO_2_ level.

## Discussion

Corals have declined and seaweeds have increased in abundance on reefs world-wide^1^,^2^,^3^,^7^,^9^,^10^. The relative roles of seaweeds damaging corals directly versus simply colonizing coral skeletons following their death from other causes is unclear and variable^6^, but numerous experimental manipulations indicate that an increase in macroalgae can directly suppress coral growth, survivorship, and recruitment^12^,^42^. As corals decline and macroalgae increase, coral-macroalgal contact increases in frequency^43^, making it critical to understand the mechanisms determining the outcome of coral-macroalgal competition^6^,^25^,^26^,^34^,^36^,^38^.

All three macroalgae we investigated induced considerable coral tissue loss and two were allelopathic to *Acropora intermedia* under some conditions, but *C. cervicornis* became allelopathic only under conditions of OA (Fig. 3, Table 2). This increase in allelopathic potency was modest (about a 5% greater suppression of PSII), but occurred after only 24 h of exposure, using only surface extractions, and would be expected to increase in effect over longer durations. A strengthening of macroalgal competition against reef-building corals under enhanced OA was documented previously for a single macroalgal species (*Lobophora cf. papenfussii*), but the mechanism was unknown and it was unclear if the cause was enhanced algal allelopathy, altered role of DOC, enhanced coral susceptibility, or a combination of processes^15^. Here we document for the first time that enhanced allelopathy with increasing OA can be a mechanism by which some macroalgae cause higher levels of coral damage under elevated concentrations of CO_2_. Investigations have documented macroalgal allelopathy against corals in about 75% of the 40 + coral-macroalgal combinations assayed to date^22^,^24^,^38^,^44^,^45^, and elevated effects of macroalgae on corals have been seen for two of the four contrasts tested under conditions of OA [i.e. *C. cervicornis* (Fig. 3), and *L.cf. papenfussii*^15^). Thus, macroalgal allelopathy against corals is common; if enhanced allelopathic potency under OA occurs in *Canistrocarpus* (=*Dictyota*), and likely in *Lobophora*^15^, which are widespread and abundant globally^3^,^46^,^47^,^48^,^49^,^50^, corals will experience greater stress from macroalgal competition as OA accelerates.

Surface extracts from the brown macroalga *C. cervicornis* grown under CO_2_ enriched conditions in flow through tanks caused greater damage to *in-situ* corals than extracts from *C. cervicornis* grown under ambient CO_2_ in equivalent flow through tanks. Because the extract experiment was conducted using *in situ* corals that had not been exposed to elevated CO_2_, CO_2_ effects on the coral can be ruled out. The stronger allelopathy noted in Fig. 3 was due to *C. cervicornis* becoming more allelopathic rather than the coral becoming more susceptible. The magnitude of the stress caused by the algal extracts in our experimental coral (quantified as a decline in fluorescence levels, EQY, Fig. 3) is smaller than that observed in corals in other coral-algal competition experiments^22^,^25^,^38^. It is likely that different coral species used in these studies have different susceptibilities to algal contact [(e.g. *A. prolifera* was more resistant to algal allelopathy than *A. millepora* using same exposure times in pilot studies conducted by the authors GDP, MEH (unpubl. data)], or that the use of different methodologies and exposure times to algal interactions causes variable damage to the corals (e.g. exposure times > 24 hrs would have caused higher coral damage). Importantly, however, coral fluorescence levels were significantly lower when exposed to extracts from *C. cervicornis* grown under high CO_2_ levels, compared to extracts from algae grown under ambient CO_2_, indicating a reduction in the photosynthetic efficiency of the coral symbionts due to lipid-soluble surface extracts alone.

Increased atmospheric CO_2_ generally enhances production of secondary metabolites in terrestrial plants^29^,^51^. Effects of elevated CO_2_ on secondary metabolites in marine plants appear variable. For example, concentrations of polyphenolics decline while concentrations of dimethylsulfoniopropionate (DMSP) appear to increase with elevated CO_2_^31^,^32^,^52^. In these examples, both phenolics and DMSP are more polar (i.e. more water soluble) secondary compounds that can mediate trophic interactions. In our experiment, we extracted lipid-soluble constituents from the surface of macroalgae because surface associated lipids have been identified as the active components in contact competitive interactions with reef corals, and have been shown to cause bleaching, suppression of photosynthesis, and sometimes mortality of corals^22^,^38^,^44^. The allelopathic compounds identified earlier^38^,^44^ are hydro-phobic terpenes associated with macroalgal surfaces. Production of some terpenes increases under elevated CO_2_ in some terrestrial plants due to higher availability of carbon for plant metabolism^53^,^54^. Elevation of CO_2_ might have enhanced production of allelopathic lipids in *C. cervicornis*, but not in the other two macroalgae we investigated (in line with^33^) (Fig. 3). Because *Ch. fastigiata* and *C. cervicornis* both make bioactive terpenes^37^,^38^,^39^, the response appears to vary by species and not simply by the class of compound involved. Enhanced concentrations of allelopathic compounds could have also occurred due to reduced macroalgal growth (as suggested for terrestrial plants^53^), or changes in other processes not considered in this study. However, reduced macroalgal growth is unlikely as parallel experiments showed the photosynthetic efficiency of our experimental *Canistrocarpus* did not change with CO_2_ (One-Way ANOVA, n = 12, F = 0.69, p = 0.55) (see Supplementary Fig. S1), and our own previous work on a closely related Dictyotaceae macroalga (*Lobophora*)^15^ and that of others on *Dictyota* has demonstrated increased growth under OA conditions^17^,^18^. The mechanisms by which elevated CO_2_ modifies the potency of *Canistrocarpus* allelopathy, or the chemical compounds involved in these interactions require additional investigation.

In our outdoor mesocosm experiment, the rates of tissue loss for corals in contact with *A. glomerata* and *Ch. fastigiata* also increased under OA conditions, but surface extracts from these species grown under elevated CO_2_ did not show enhanced allelopathic activity on corals *in situ*. Thus, other competitive mechanisms or some processes affecting coral physiology may be driving interactions among these species. Corals exposed to OA but free of macroalgae experienced some tissue loss, suggesting that elevated CO_2_ alone is a physiological stressor^19^, and this stress may have made the corals in our experimental tanks more susceptible to algal effects (including allelopathic effects) and space competition than the *in situ* corals we used for testing allelopathic potency (Fig. 3)^55^,^56^. In addition to producing allelopathic lipids, macroalgae may also stress corals indirectly by releasing dissolved organic carbon (DOC), which may alter microbial community composition and the balance between beneficial versus pathogenic microbes on coral surfaces and lead to coral death^25^,^34^,^47^. Little is known about the effects of elevated CO_2_ on DOC production by macroalgae, but it is possible that macroalgae under conditions of OA may uptake excess CO_2_ and increase release of DOC, as suggested for planktonic microalgae^57^. Increased release of DOC could have contributed to coral tissue loss in our experimental tank experiment (Figs 1 and 2), but could not explain the allelopathy seen in our field assays using gel-strips (Fig. 3) because these contained the non-polar, not the polar, extracts from macroalgal surfaces. A combination of enhanced coral sensitivity to macroalgal allelochemicals, shifts in microbial communities, and DOC release may have acted in concert to compromise coral health in the presence of *A. glomerata* and *Ch. fastigiata* (and possibly *C. cervicornis*) under elevated CO_2_. Our results show that although all experimental macroalgae caused damage to the corals when exposed to elevated CO_2_ levels, there is variability in the mechanisms by which algae stress and kill corals, suggesting that the relative importance of each mechanism varies under OA. Allelopathy in *C. cervicornis*, and possibly an altered role of DOC in *A. glomerata* and *Ch. fastigiata*, in addition to direct impacts on coral holobiont physiology and coral microbiomes may play important roles in driving coral-macroalgal interactions as concentrations of CO_2_ increase in future oceans.

Our study has implications for understanding the impacts of OA on the dynamics of coral-macroalgal interactions, which may cascade to affect coral-algal phase shifts. First, we demonstrate that common macroalgae are allelopathic to corals and that this allelopathy is strengthened for one of the three macroalgae we investigated under elevated CO_2_. This suggests that the competitive advantage of some macroalgae over corals may increase and further accelerate phase shifts as OA continues. This may be exacerbated by enhanced macroalgal growth and cover under projected near-future CO_2_^15^,^16^,^17^,^18^,^20^,^58^. Secondly, our data demonstrate variance in competitive mechanisms and intensity between coral and macroalgae under elevated CO_2_ levels. For example, the brown alga *C. cervicornis* and the green alga *Ch. fastigiata* both damage corals faster (Fig. 2) than the red alga *A. glomerata. C. cervicornis* and *Ch. fastigiata* both produce potent allelochemicals^22^,^38^,^43^. *A. glomerata* is not known to produce strong, bioactive compounds that damage corals (although it may be allelopathic against bacteria^59^), and it affects corals more slowly. This variability implies that coral reefs dominated by Dictyotaceae algae like *C. cervicornis* and *Lobophora papenfussii*^15^, may be more susceptible to damage than reefs dominated by *A. glomerata* as CO_2_ concentrations increase. The macroalga *Ch. fastigiata* is common but rarely abundant on reefs^43^ and may be a lesser concern under future OA scenarios.

Our results may be particularly important given the current dominance of *Dictyota* (now changed to *Canistrocarpus* for some species) and *Lobophora* on many Caribbean^3^,^23^,^48^ and a number of Indo-Pacific reefs^49^. The dominance of these Dictyotaceae seaweeds has been attributed mainly to reductions of grazing by reef herbivores and to additional space made available by coral mortality from cyclones, disease, thermal bleaching, and crown-of-thorn-starfish^49^,^60^,^61^. Dictyotaceae seaweeds, however, may also actively damage corals via a variety of direct (e.g. overgrowth and allelopathy^22^,^24^) and indirect (e.g. DOC release and alteration of coral microbial activity and coral microbiome^26^,^28^) mechanisms (or combination of both, e.g. ref. 26), contributing to coral decline and macroalgal dominance. The increased potency of allelopathic interactions with *C. cervicornis* (and potentially *Lobophora papenfussii*^15^) due to OA conditions, suggests that the 75% increase in CO_2_ concentrations that have occurred since preindustrial times^13^ may already be advantaging these macroalgae over corals. Dictyotaceae species such as *Dictyota*^21^, *Padina*^62^ and *Spatoglossum*^63^ are known to proliferate in naturally acidified reefs, although whether these taxa also enhance the potency of allelopathic interactions against corals under elevated CO_2_ needs to be investigated. Further, enhancement of algal allelochemicals due to CO_2_ enrichment may not only alter competitive interactions, but also trophic interactions by reducing macroalgal palatability and consequently macroalgal removal by herbivores (via reduction in grazing), although this hypothesis needs testing (e.g. ref. 33) in Dictyotaceae algae. Reduction in herbivory pressure due to overfishing^9^ as well as enhanced coral mortality due to increased sea surface temperature^19^,^64^, declining water quality^47^, and reductions of coral recruitment due to algal competition and dominance^12^,^65^ will further contribute to algal proliferation, ultimately eroding reef resilience. Numerous seaweeds may be competitively advantaged over corals as OA increases. This will occur not only from corals being weakened by OA, but also by the allelopathic potency of some seaweeds being enhanced by OA or by other mechanisms not tested in this study (e.g. altered DOC production/consumption). This adds to the complex list of interacting stressors that tropical reefs face now, as well as in the future.

## Methods

### Effects of elevated CO_2_ on coral-macroalgal competition

To evaluate the effects of elevated CO_2_ on coral-macroalgal competition and how this varies among species pairings, we conducted a factorial outdoor experiment at the Heron Island Research Station (HIRS), GBR (23°26′30″S–151°54′46″E). We used the branching coral *Acropora intermedia* and three fleshy macroalgae, the green alga *Chlorodesmis fastigiata,* the brown alga *Canistrocarpus* (=*Dictyota) cervicornis,* and the red alga *Amansia glomerata.* These seaweeds were common and frequently in contact with corals on the reef^66^. Corals and macroalgae were collected by hand at 3–6 m depth from Harry’s-Bommie, Heron Island, GBR (23°27′32″S, 151°55′45″E) and acclimatised separately in shaded outdoor tanks using a flow-through system with ambient conditions for two weeks. Light levels in the acclimatisation tanks were similar to those of the experimental containers (see below). *A. intermedia* was the assay coral because it is abundant in the study area (average cover of 22% in the colleting site, but can be locally dominant, Del Monaco, unpubl. data). *C. cervicornis* and *A. glomerata* are also abundant in Heron reefs, the latter particularly so at the base of coral branches in coral dominated areas. Transect surveys show *C. cervicornis, A. glomerata* cover in average 4% and 5% respectively in shallow reefs of Heron Island (Del Monaco, unpubl. data). *Ch. fastigiata* is common but less abundant (1% cover), and similar to *C. cervicornis*, it produces bioactive terpenoids that harm corals^38^.

Our factorial experiment consisted of a coral-algal competition treatment with five levels and an ocean acidification treatment with three levels of CO_2_/pH manipulation. The coral-algal competition treatment included (1) coral- *Ch. fastigiata* interaction pair (a live coral branch with the alga *Ch. fastigiata* attached to the base with a plastic cable tie); (2) coral- *C. cervicornis* interaction pair (as for the previous treatment level), (3) coral- *A. glomerata* interaction pair, (4) coral control (coral alone, no alga present); and (5) a coral branch in contact with an inert algal mimic (monofilament fabric) attached to the base of the coral with a plastic cable tie; this assessed the effect of an alga’s physical, but not chemical or biological, presence on the coral (as per^15^)(see also Supplementary Fig. S2 for diagram of the experimental design). Due to logistic constrains, we could only use one type of algal mimic morphology for the three algal species. Coral branches were 8–10 cm long and the algal biomass was 3–7 g (wet weight).

Corals and macroalgae were exposed to three CO_2_ levels; ambient (380 CO_2_ ppm, pH = 8.16 ± 0.02), medium (540 CO_2_ ppm; pH = 7.86 ± 0.03), and high (936 CO_2_ ppm; pH = 7.70 ± 0.02) CO_2_, simulating current levels and those predicted for the years 2050 and for 2100, respectively, under the RCP 8.5 model of the Intergovernmental Panel for Climate Change (IPCC)^13^. CO_2_ concentrations were achieved using established methods^15^,^67^. In brief, pH was regulated by computer operated solenoid valves (Aquatronica-AEB Technologies, Cavriago, Italy) that controlled the amount of analytical grade CO_2_ bubbled into 200 l mixing sumps. pH sensors (Mettler-Toledo, InPro4501VP) logged measurements every 30 minutes, and calibration was linearly performed using three NIST-certified pH buffers (Mettler Toledo, Switzerland). Ambient and CO_2_ treated seawater from the mixing sumps continuously fed the experimental tanks (20 l plastic containers, see below) at a flow rate of 1.79 ± 0.08 (SEM) l/min using unfiltered seawater from the reef flat. Total alkalinity was estimated from each CO_2_ treatment by an open-cell potentiometric titrator (Model T50 Mettler Toledo) using triplicate water samples taken from the experimental containers every 4 hours during a 24 h-period. We also collected water samples from the sumps and did not find significant differences in carbonate chemistry between sumps and experimental tanks (Table 3, *t*-student, p = 0.95). Total alkalinity and pH values determined in the samples were utilised to constrain the carbonate chemistry of the experimental system^68^,^69^ (Table 3).

Three replicate 20 l tanks were used for each combination of competition, CO_2_, and macroalgal treatment levels, each tank holding four experimental sub-samples (see Supplementary Fig. S2). Species of macroalgae were not intermixed in each tank but kept separate. Coral control and coral-algal mimic levels were the same for the three algal species and due to logistic constrains, no attempt was made to mimic individual algal morphology. Sub-samples were randomly allocated to replicate tanks and were suspended in the water column on a fishing line. A small power head in each tank assured water circulation, and all tanks were cleaned every 3 days to reduce biofilms. Shading screens were placed over the experimental area to reduce light intensity and heating, approximating field conditions. Underwater light measurements in the tanks averaged 242.11 ± 84.78 (SEM) μM at 4:00 pm (*in situ* light measurements in the field ranged between 220 and 488 μM at 4 m depth at 4:00 pm; all light measurements were obtained using a LI-1400 data logger, LI-192SA sensor). Seawater temperature averaged 25.87 ± 0.14 (SEM) °C and salinity was 34.46 ppm (measured using a portable refractometer) during the experiment. The experiment was carried out for 25 days during March 2013. The response variable included a measurement of coral tissue loss (an indicator of partial colony mortality), which was recorded daily in the tanks as the amount of sloughed tissue using four categories: 0, 1–33, 34–66 and 67–100% per coral branch. Coral branches exhibiting partial tissue loss were examined under the stereomicroscope confirming the absence of coral tissue in the affected areas. Coral tissue loss generally progressed from the base upwards along the branch.

### Effects of elevated CO_2_ on the potency of macroalgal allelopathy

To test whether elevated CO_2_ enhanced the allelopathic potency of seaweeds, the macroalgae *C. cervicornis, A. glomerata and Ch. fastigiata* were exposed to elevated CO_2_ concentrations, their surface metabolites extracted, these extracts applied to corals *in situ,* and the damage on the corals compared to control extracts from algae grown at ambient CO_2_ concentrations. Macroalgal collections and CO_2_ manipulations were conducted in the same way as described above. Macroalgae were exposed to two levels of CO_2_, ambient (control, pH: 8.16 ± 0.02 SEM) and high (936 CO_2_-ppm, pH: 7.69 ± 0.02 SEM) for 14 days (February 25^th^ to March 10^th^, 2013). Ambient and high CO_2_ treated seawater from the mixing 200 l sumps continuously fed the experimental tanks at a flow rate of 1.79 l/min (see sumps carbonate chemistry data in Table 3). Each macroalgal species was grown separately in 200-l plastic sumps exposed to natural sunlight. Experimental conditions including light levels, temperature, and water circulation were the same as for the previous experiment. Following the two week CO_2_ treatment, surface allelochemicals from the experimental macroalgae were extracted following existing protocols^70^. In short, approximately 20 ml displacement volume of each algal species (which included several individuals) was cleaned of fouling organisms and spun with a salad spinner to remove excess water; algae were then submerged in hexane (98%) and vortexed for 30 seconds. Algal tissue was removed, the solvent removed via rotary evaporation, and the hexane-soluble surface extract retained, and incorporated at natural volumetric concentration into 1 × 1 cm Phytagel pads (following methods of^22^,^38^). Previous assays with allelopathic seaweeds showed this method was entirely sufficient to obtain bioactivity from the algal surface that equalled activity of the entire extract^22^,^38^,^44^. Gel pads impregnated with macroalgal extracts from each species and CO_2_ level were put into contact with the surface of *A. intermedia* branches (n = 15) *in situ* at a depth of 6 m on the fore reef of Heron Island (Harrys Bommie). Gel pads were placed approximately 3–5 cm from the tip of the coral branch and gently secured with a plastic cable tie. 15 replicates per CO_2_ and algal treatment combination were used in addition to 15 control pads (same solvent used but no extracts, i.e. Phytagel controls) to test for possible effects of the gels, solvents, and extraction protocol on the corals. Each coral branch contained only one gel and gel pads from the different algal species were not intermixed in each coral branch. In total, we used 105 gel pads that were placed on coral colonies distributed over an area of approx. 150 m^2^. Gel pads were removed after 24 hours and the symbiotic dinoflagellate chlorophyll fluorescence (Effective Quantum Yield, EQY) assessed *in situ* beneath the area that had been covered by the gel pad using a Pulse-Amplitude-Modulation Fluorometer [Walz, Model: Diving-PAM with red measuring light LED, 650 nm; settings: damp = 2 and gain = 1–3; fiberoptics: DIVING-F, active diameter 5.5 mm; all measurements (one per coral branch) were made at a distance of 2 mm from the coral surface and to standardise that distance we used a plastic tube adapter]. EQY has been previously used to estimate photosynthetic efficiency and coral health^38^,^41^. The 24 hour exposure period to the gel pads was insufficient to induce coral tissue loss or considerable bleaching (authors’ per. obs.), therefore EQY was chosen for this experiment. All PAM measurements were conducted between 11:00 and 13:00 hrs, with treatment types interspersed through time.

### Data analyses

The rate of coral tissue loss was calculated on a daily basis, and values averaged by the number of days until complete loss of coral tissue, or until the end of the experiment at day 25 (if living tissue remained at the end of the experiment) (Fig. 1). This rate of tissue loss was statistically compared among treatments with a two-way nested ANOVA, with CO_2_ and coral-algal competition as fixed factors, tanks as the nested component, and branches as replicates. There was no significant effect of the nested (i.e. tank) component on the rate of coral tissue loss (p = 0.584). We therefore pooled the data to increase the power of the analyses^71^ and subsequently conducted a two-way (non-nested) ANOVA with 12 replicate branches per treatment combination, followed by *post hoc* Tukey tests (Table 1).

EQY values of corals were compared with a two-way ANOVA with CO_2_ (two levels: extracts of algae grown under ambient CO_2_, and extracts from algae grown under high CO_2_) and algal treatments (three algal species) as fixed factors. The analyses included 15 coral branches as replicates. Significant interactions occurred between treatments, therefore we conducted subsequent one-way ANOVAs within treatment combinations followed by *post hoc* Tukey tests. EQY values for each CO_2_ and algal treatment combination were also compared against the phytagel control using a 2-sided Dunnett test using SYSTAT software. Data were tested for normality using Shapiro-Wilk test and for homogeneity of variances using Levene-Equality of Several Variance (for fluorescence values) and Cochran tests (for coral tissue loss) and data were not transformed for the analyses.

## Additional Information

**How to cite this article**: Del Monaco, C. *et al*. Effects of ocean acidification on the potency of macroalgal allelopathy to a common coral. *Sci. Rep.* **7**, 41053; doi: 10.1038/srep41053 (2017).

**Publisher's note:** Springer Nature remains neutral with regard to jurisdictional claims in published maps and institutional affiliations.

## Supplementary Material

Supplementary Information

## Figures and Tables

**Table 1:** Two-way ANOVA to test for the effects of CO_2_ and coral-macroalgal competition on the rate of coral tissue loss per day (Fig. 1).

Source of variation	df	MS	F	p-value	Conclusion (Tukey Test)
CO_2_	2	234.6	35.2	<0.001	High = Med > Amb
Algal competition (AC)	4	267.9	40.2	<0.001	Can > Chlo = Ama > Mimic = Control
CO_2_*AC	8	3.5	0.5	0.840	
Error	164	6.7			

CO_2_ levels included: ambient (Amb), medium (Med), and high. Coral–macroalgal competition levels included: coral with the algae *Canistrocarpus* (=*Dictyota) cervicornis* (Can), *Chlorodesmis fastigiata* (Chlo), and *Amansia glomerata* (Ama), coral without algae (coral control = Control) and coral with an inert algal mimic (Mimic). df: degrees of freedom; MS: mean squares; F: test statistic.

**Table 2:** Two-way ANOVA to test for the effects of extracts from macroalgae [*Canistrocarpus* (=*Dictyota) cervicornis* (Can), *Chlorodesmis fastigiata* (Chlo), and *Amansia glomerata* (Ama)] grown under two CO_2_ levels [ambient (Amb) and high] on effective quantum yield (EQY) of *Acropora intermedia* corals (as a measure of the potency of allelopathic extracts; Fig. 3).

Source of variation	df	MS	F	p-value	Conclusion (Tukey Test)
CO_2_	1	<0.001	0.111	Can: 0.037	Can: Ambient > High
				Chlo: 0.775	Chlo: ns
				Ama: 0.074	Ama: ns
Algae	2	0.006	3.511	Ambient: 0.078	Ambient: ns
				High: 0.025	High: Ama > Can = Chlo
CO_2_*Algae	2	0.005	3.073	0.052	
Error	164	6.7			

**Table 3:** Seawater carbonate chemistry parameters estimated in experimental tanks and mixing sumps for each CO_2_ level in both experiments.

CO_2_ level	Temp (°C)	pH NBS/NIST	pCO_2_ (ppm)	DIC (μmol/KgSW)	TA (μmol/Kg SW)	ΩArag
High						
Tank	25.58	7.70	936	2109.95	2245.28	1.52
	(0.58)	(0.02)		(13.86)	(1.65)	(0.04)
Sump	25.58	7.70	936	2108.72	2243.99	1.51
	(0.59)	(0.03)		(11.87)	(2.71)	(0.01)
Medium						
Tank	25.58	7.86	540	2036.55	2241.37	2.13
	(0.58)	(0.03)		(13.77)	(2.06)	(0.05)
Sump	25.58	7.86	540	2036.34	2241.15	2.11
	(0.59)	(0.04)		(12.57)	(8.41)	(0.02)
Ambient						
Tank	25.58	8.16	380	1862.20	2236.27	3.74
	(0.58)	(0.02)		(12.09)	(3.96)	(0.08)
Sump	25.58	8.16	380	1865.6	2240.11	3.74
	(0.59)	(0.03)		(12.45)	(4.65)	(0.02)

PCO_2_ = partial pressure of CO_2_; DIC = Total dissolved inorganic carbon; TA = Total alkalinity; Ω_Arag_: Aragonite saturation state. Salinity of 34.46 was used for the calculations using the CO2SYS computer software. Values are means [n = 12 (3 samples collected every 4 hours during a 24 hr period)] ± SE.
