# Supplementary material for: Effects of ocean acidification on the potency of macroalgal allelopathy to a common coral
Source: Sci Rep. 2017 Feb 1;7:41053. doi: 10.1038/srep41053 (PMC5286515; doi:10.1038/srep41053)
Supplement: Supplementary Information [file srep41053-s1.pdf]

Supplementary Information

Effects of ocean acidification on the potency of macroalgal allelopathy to a common coral

Authors: Carlos Del Monaco, Mark E. Hay, Patrick Gartrell, Peter J. Mumby and Guillermo Diaz-Pulido

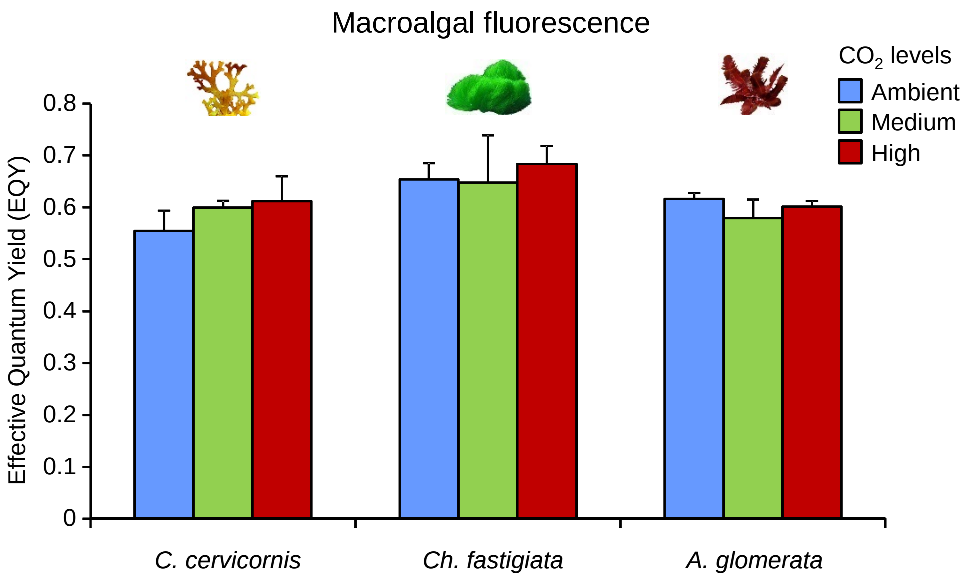

Supplementary Figure S1. Fluorescence values (effective quantum yield) of the macroalgae *Canistrocarpus (=Dictyota) cervicornis*, *Chlorodesmis fastigiata* and *Amansia glomerata* grown under three levels of CO_2: ambient (380 ppm); medium (540 ppm) or high (936 ppm). Experiment was conducted in parallel with the first experiment. One-way ANOVA showed no significant effects of CO_2 (n=12, F=0.69, p=0.55).

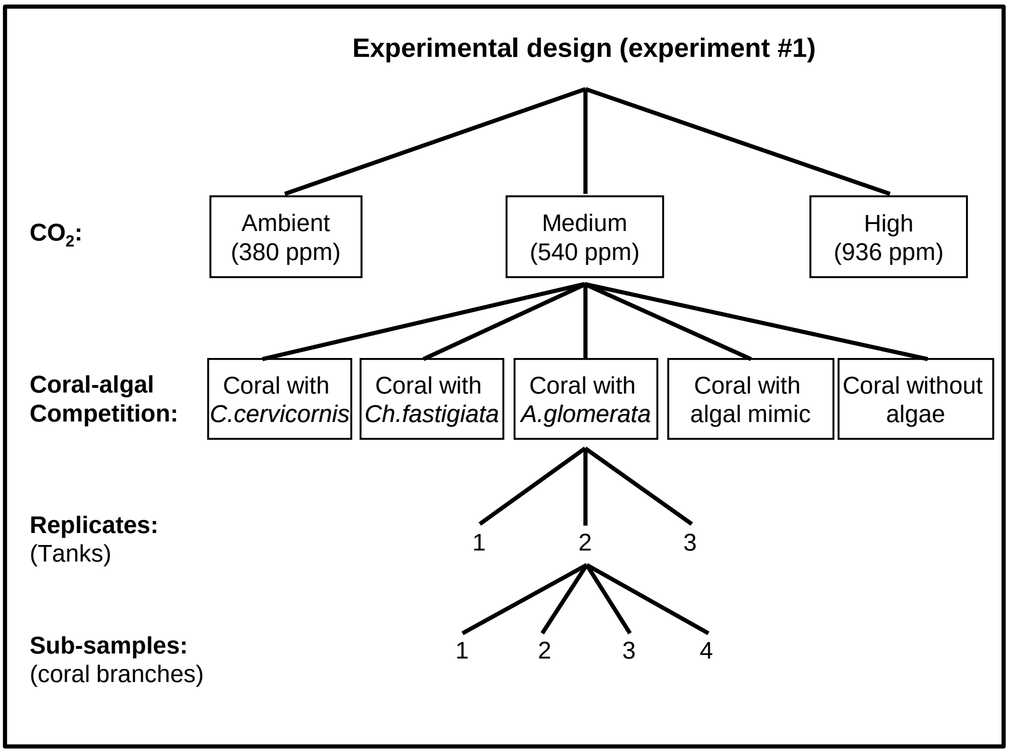

Supplementary Figure S2. Diagram of the Experimental design used to generate Fig. 1.
